# Sch-net: a deep learning architecture for automatic detection of schizophrenia

**DOI:** 10.1186/s12938-021-00915-2

**Published:** 2021-08-03

**Authors:** Jia Fu, Sen Yang, Fei He, Ling He, Yuanyuan Li, Jing Zhang, Xi Xiong

**Affiliations:** 1 College of Biomedical Engineering, Sichuan University, Chengdu, China; 2 Mental Health Center, West China Hospital of Sichuan University, Chengdu, China; 3 School of Cybersecurity, Chengdu University of Information Technology, Chengdu, China

**Keywords:** Schizophrenia, Deep learning, Skip connection, Attention mechanism, Pathological speech detection

## Abstract

**Background:**

Schizophrenia is a chronic and severe mental disease, which largely influences the daily life and work of patients. Clinically, schizophrenia with negative symptoms is usually misdiagnosed. The diagnosis is also dependent on the experience of clinicians. It is urgent to develop an objective and effective method to diagnose schizophrenia with negative symptoms. Recent studies had shown that impaired speech could be considered as an indicator to diagnose schizophrenia. The literature about schizophrenic speech detection was mainly based on feature engineering, in which effective feature extraction is difficult because of the variability of speech signals.

**Methods:**

This work designs a novel Sch-net neural network based on a convolutional neural network, which is the first work for end-to-end schizophrenic speech detection using deep learning techniques. The Sch-net adds two components, skip connections and convolutional block attention module (CBAM), to the convolutional backbone architecture. The skip connections enrich the information used for the classification by emerging low- and high-level features. The CBAM highlights the effective features by giving learnable weights. The proposed Sch-net combines the advantages of the two components, which can avoid the procedure of manual feature extraction and selection.

**Results:**

We validate our Sch-net through ablation experiments on a schizophrenic speech data set that contains 28 patients with schizophrenia and 28 healthy controls. The comparisons with the models based on feature engineering and deep neural networks are also conducted. The experimental results show that the Sch-net has a great performance on the schizophrenic speech detection task, which can achieve 97.68% accuracy on the schizophrenic speech data set. To further verify the generalization of our model, the Sch-net is tested on open access LANNA children speech database for specific language impairment detection. The results show that our model achieves 99.52% accuracy in classifying patients with SLI and healthy controls. Our code will be available at https://github.com/Scu-sen/Sch-net.

**Conclusions:**

Extensive experiments show that the proposed Sch-net can provide aided information for the diagnosis of schizophrenia and specific language impairment.

## Background

Psychological and neurological disorders are two major categories of human disorders, which affect the thinking, speaking, and behavior capacity of human beings [1, 2]. At present, the global prevalence of psychological and neurological disorders is more than 12% and 10%, respectively [1–4]. Schizophrenia is a chronic psychological disease that affects about 1% of the population worldwide [5, 6]. The disease often begins in late adolescence, and it has a large impact on patients’ social activity and brain development. Schizophrenia is characterized by disordered thinking, impaired speech, and abnormal behaviors. Clinical diagnosis of schizophrenia is generally based on a full psychiatric assessment and the speech/behaviors observed via clinical interviews. Symptoms of schizophrenia can be divided into two types, positive symptoms, and negative symptoms. Positive symptoms include delusions and hallucinations [6, 7], and negative symptoms include flat affect, alogia, loss of interest, and disability in activities [8]. Clinical experience had shown that it is harder to diagnose and treat patients with negative symptoms than those with positive symptoms [9]. Positive symptoms are likely to be replaced by negative symptoms in the late episode of schizophrenia, and negative symptoms may persist even though after treatment [10]. Negative symptoms contribute more to the long-term morbidity, higher rates of disability, and poor quality of life in most schizophrenic patients than positive symptoms do [11–15]. In addition, the clinical diagnosis relies on the experience of clinicians and is affected by patients’ retrospective recall biases and cognitive limitations [16]. Hence, it is urgent to propose a method to diagnose schizophrenic patients with negative symptoms objectively and effectively.

Patients with schizophrenia exhibit brain structural abnormalities [17–19], which are accountable for speech disorders and cognitive impairments. Cohen [20] discovered that speech characteristics are significantly related to the negative symptoms of schizophrenia. Rosenstein [21] confirmed that adolescents with high-risk psychosis exhibit speech impairments for months/years before they are diagnosed. Flat affect and incoherent language expression are typical performances in schizophrenic patients with negative symptoms [22]. Schizophrenic groups exhibit reduced pitch variation [23], increased pauses [24], and poverty of content [25]. The number and duration of pauses are closely related to the evaluation of affective flattening [8, 26, 27].

In general, most existing methods [16, 28–38] analyzed schizophrenic speech using feature engineering techniques, which were achieved by extracting fluency features, intensity-related features, spectrum-related features, and so on. These studies had proved that speech can be viewed as an automated biomarker for the diagnosis of schizophrenia. However, owing to the limitation in the amount of data and the difficulties in effective feature extraction, it is still difficult to propose a robust model. In this work, the Schizophrenia network (Sch-net) based on a convolutional neural network (CNN) is proposed to achieve the end-to-end schizophrenia detection based on speech signals. The proposed Sch-net can avoid the problems of feature extraction. The contributions of our work can be summarized as follows:

1. This work proposes the Sch-net to detect schizophrenia based on speech signals. To the best of our knowledge, this is the first work to detect schizophrenic speech using CNN-based architecture.
2. The proposed model adds the skip connection to the backbone network. It enriches the information via merging low-level feature maps with high-level feature maps, which avoids the manual feature extraction procedure.
3. The proposed model utilizes the convolutional block attention module (CBAM). The CBAM performs the automatic feature selection function by giving learnable weights to the features in the feature maps.
4. The proposed Sch-net is validated on the schizophrenic speech data set and specific language impairment (SLI) speech database. Experimental results have demonstrated that our method can provide aids for the diagnosis of schizophrenia and SLI.

## Related works

The detection of disordered speech in schizophrenia has been studied for the last few decades. Previous studies [16, 28–38] are mainly achieved based on feature engineering. In this section, we will review the related studies from the perspective of features. The features extracted can be roughly divided into two categories, time-domain features, and spectrum-related features.

Time-domain features: Schizophrenic patient with negative symptoms usually exhibits incoherent language that can be described by time-domain features, including pitch-related features, fluency features, and intensity-related features. (1) *Pitch-related features:* Pitch is the fundamental frequency of vocal cord vibration for voiced initial consonants and some unvoiced initial consonants [39]. Pitch-related features are commonly used in analyzing the flat affect in schizophrenia. [16, 28–34]. Studies [28, 30–32] demonstrate that schizophrenic speech is characterized by less variability in vocal pitch than normal speech. (2)* Fluency features:* The incoherent expression in schizophrenia usually manifests as more pauses and a longer duration of pauses. Fluency features are employed to distinguish schizophrenic groups and controls in recent studies [30, 35, 36], such as the number of pauses and natural turns, the duration of pauses, the proportion of silence and speaking, and speaking rate. (3) *Intensity-related features:* Voice intensity is an intuitive indicator for conveying emotional information in human communication [40]. Previous studies [28, 30, 32] calculate the intensity-related features based on the variability of energy per second/syllable, and the experimental results demonstrate that the voice intensity of patients with schizophrenia has less variation than that of controls.

Spectrum-related features: Spectrum-related features generally refer to the measurements computed based on the spectrum that contains time- and frequency-domain information. Spectrum-related features describe the energy distribution and the vocal tract characteristics during speech production. The typical spectrum-related features, such as formants, auditory-based spectral features, and spectral envelope features, have been proven to be effective for schizophrenia detection [32, 33, 37, 38]. (1) *Formants:* Formant is the descriptor that reflects the resonance frequency of the vocal tract. Compton et.al [32] demonstrate that the range of the second formant for schizophrenic speech is smaller than that for controls. Chhabra et.al [37] conclude that patient with schizophrenia reduces the use of formant dispersion in the similarity-dissimilarity ratings. (2) *Auditory-based spectral features and spectral envelope features:* Auditory-based spectral features refer to the spectral parameters that are computed based on human auditory characteristics, and spectral envelope features refer to the envelope and its variants of the spectrum. Mel-frequency cepstral coefficient (MFCC) is one typical auditory-based spectral feature, and linear prediction coefficient (LPC) is a commonly used spectral envelope feature. MFCC is gained using Mel-frequency filters, in which the center frequency is computed according to the human auditory characteristics. LPC is calculated to estimate the resonance characteristics of the vocal tract during speech production. Studies [33, 38] use MFCCs and LPCs to analyze the characteristics of schizophrenic speech. Results in [38] show that the MFCC and LPC scores of schizophrenic speech are significantly lower and higher than those of controls, respectively.

Low-level acoustic features mentioned above [28–38] are generally extracted using OpenSMILE, pyAudioAnalysis, openEAR, and signal processing techniques. Classification experiments are conducted using classifiers (such as k-Nearest Neighbors, Decision Trees, Naive Bayes), combined with cross-validation (such as k-fold cross-validation and leave-one-out cross-validation). Studies [30, 32–36] have achieved 64–93% accuracy on schizophrenia detection tasks using 8–98 schizophrenic patients and 7–102 controls.

## Results

To demonstrate the effectiveness of the proposed model, comprehensive experiments are conducted. We first describe the schizophrenic speech data set and implementation details. Next, the ablation studies are presented to demonstrate the advantages of each component in the proposed Sch-net. Then comparisons with state-of-the-art methods based on feature engineering and deep learning techniques are conducted and analyzed. The network visualization is also presented using Grad-CAM. Finally, to further validate the generalization of proposed method, the classification experiments on the LANNA children speech database are conducted.

### Schizophrenic data set

Our study has 28 schizophrenic patients (18 females and 10 males) and 28 matched healthy controls (18 females and 10 males). The schizophrenic group is with a mean age of 40.6 years (SD 9.4 years), and the control group is with a mean age of 36.5 years (SD 9.1 years). All subjects are native Mandarin speakers, and they have no past or current disease affecting the speaking process. Patients were recruited from the Psychiatry Department of the Mental Health Center, Sichuan University. This department is one of the four major mental health centers in China. The schizophrenic group was diagnosed by clinicians based on the Diagnostic and Statistical Manual of Mental Disorders, Fifth Edition (DSM-5) that outlines the concise and explicit criteria for the diagnosis of schizophrenia [41]. All subjects provided the written informed consent.

The data set is composed of audio signals that are recorded in a 16-bit mono/dual-format at a sampling rate of 44.1kHz. Participants are asked to achieve the reading task. There are four texts with calm, happiness, anger, and fear sentiments, and each text comprises 8–10 sentences. We select a fixed sentence for each emotional recording, and the transcriptions of speech signals are listed in Table 1.

**Table 1:** Text for speech recording in Mandarin and its corresponded English translation

Emotion	Text (Mandarin)	Text (English)
Calm	Ta yi nian si ji dou ke yi kai hua, hua duo yi ban shi hong se huo fen se de.	It can bloom all year round, and the flowers are generally red or pink.
Anger	Gen ni shuo le duo shao ci le, bu xu wan wo de wan! Kan ba, wan bei da sui le! Ni zhen de shi yao qi si wo!	I told you so many times that you are not allowed to play with my bowls! Look, the bowl is shattered! You are really mad at me!
Fear	Ma ma, dui bu qi, wo...wo...wo bu shi gu yi de.	Mom, I’m sorry, I...I...I didn’t mean it!
Happiness	Ha ha, tai hao la! Tai hao la! Ma ma, ma ma, wo kao le 98 fen!	Awesome, it’s awesome! Mom, Mom, I got 98 points!

### Implementation details

In this study, all audios are converted to spectrograms using the Short-time Fourier Transform (STFT) method. To improve the invariance properties to geometric perturbations and noise, data augmentation methods are utilized, including random crop, random rotation, random rescaling, random Gaussian noise, masking blocks of frequency channels [42], and masking blocks of time steps [42].

The input image of the Sch-net is with the size of 128$\times$256 pixels. Table 2 shows the Sch-net architecture details. In this architecture, the size of each filter in Conv layers is set as 3 $\times$ 3. There are 64, 128, 256, 512 filters in the first to the fourth Conv layers, respectively. In addition, there are 512 filters in the three skip connections. The convolved images are normalized using a ReLU activation in Conv blocks. The max pooling and average pooling in pooling layers are obtained every 2 $\times$ 2, with a stride of 2. In the CBAM, 2048 filters of size 7 $\times$ 7 are used to highlight effective features. The highlighted features are convolved with 512 filters of size 3 $\times$ 3. In the FC neural network, there are 512 neurons in the first hidden layer and 2 neurons in the second layer. The final output is a vector of probabilities that the input sample will belong to each class.

**Table 2:** Sch-net architecture details

Layer	Dimension
Conv1	2×[3×3(64 filters)]
Conv2	2×[3×3(128 filters)]
Conv3	2×[3×3(256 filters)]
Conv4	2×[3×3(512 filters)]
Conv5-8	3×3(512 filters)
Max-pooling	2×2
Average-pooling	2×2
CBAM	7×7 (2048 filters)
FC	1×1×512, 1×1×2 (two hidden layers)

In all experiments, the binary cross-entropy is adopted as the loss function, and Adam [43] is used as the optimization algorithm. All experiments are implemented based on the PyTorch framework [44] and trained on a workstation with Intel(R) Xeon(R) CPU E5-2680 v4 2.40 GHz processors and an NVIDIA Tesla P40 (24 GB) installed. The network is trained using batch size 16 for 50 epochs. The initial learning rate is set to 0.0003 and decreases by 10 times after 25 epochs. $\cdot$

### Ablation studies

**Fig. 1:**
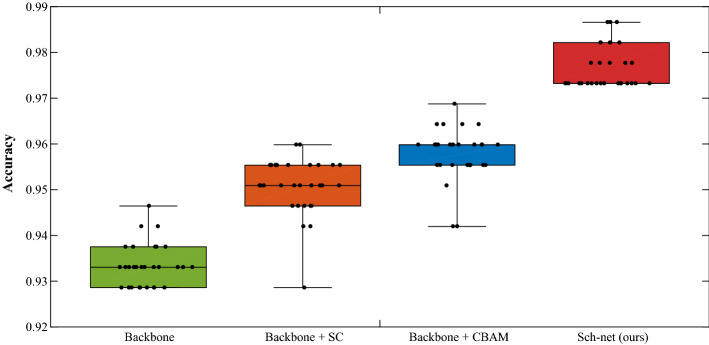
Box plots of accuracy for classifying schizophrenic speech and controls using Sch-net and its components (backbone, skip connection (SC), and CBAM)

In this subsection, the effectiveness of our network is verified. The Sch-net’s backbone network is based on CNN, with adding skip connections to enrich the feature information. In addition, the CBAM is applied to emphasize the more effective features with bigger weights. For this ablation study, we evaluate the contributions of the two key components to discriminate schizophrenic patients from healthy controls. To evaluate the performance of Sch-net and its components (backbone, skip connection, and CBAM), we run 30 iterations of tenfold cross-validation and compute seven metrics (accuracy, precision, recall, f1-score, sensitivity, specificity, and Area Under ROC Curve (AUC)) for each model. The 95% Confidence Intervals (CIs) for the metrics are listed in Table 3, and the box plots of classification accuracies are shown in Fig. 1.

**Table 3:** Overall performance of schizophrenic speech detection using Sch-net and its components (backbone, skip connection (SC), and CBAM)

Evaluated indicators	95% CI			
	Backbone	Backbone + SC	Backbone + CBAM	Sch-net (ours)
Accuracy	0.9323	0.9494	0.9563	0.9768
	(0.9295,0.9351)	(0.9460,0.9528)	(0.9534,0.9591)	(0.9739,0.9797)
Precision	0.9480	0.9634	0.9513	0.9639
	(0.9445,0.9515)	(0.9564,0.9704)	(0.9458,0.9568)	(0.9585,0.9693)
Recall	0.9149	0.9348	0.9622	0.9908
	(0.9100,0.9197)	(0.9326,0.9370)	(0.9556,0.9688)	(0.9898,0.9918)
F1-score	0.9311	0.9487	0.9565	0.9771
	(0.9280,0.9341)	(0.9456,0.9519)	(0.9536,0.9594)	(0.9743,0.9799)
Sensitivity	0.9176	0.9619	0.9902	0.9914
	(0.9131,0.9221)	(0.9581,0.9657)	(0.9847,0.9956)	(0.9863,0.9964)
Specificity	0.9488	0.9601	0.9494	0.9738
	(0.9415,0.9561)	(0.9513,0.9689)	(0.9437,0.9551)	(0.9656,0.9820)
AUC	0.9593	0.9892	0.9902	0.9978
	(0.9577,0.9609)	(0.9859,0.9924)	(0.9880,0.9924)	(0.9965,0.9990)

In each box plot in Fig. 1, there are five points (the median, the upper and lower quartiles, and the minimum and maximum values) to display the distribution of classification accuracies for each model. As can be seen in Table 3 and Fig. 1, the skip connection enriches the information of feature maps and improves the classification accuracy by 1.71% on the schizophrenic speech data set. The CBAM selects the meaningful features for classification and improves accuracy by 2.40%. Significant improvement of 4.45% for classifying schizophrenic speech and normal speech is achieved when adding skip connections and CBAM to the backbone network. The proposed Sch-net combines the advantages of skip connection and CBAM, achieving better performance on the classification task.

### Comparison with the models based on feature engineering and classifiers

Previous studies about automatic schizophrenic speech detection [28–38] are almost based on feature engineering and pattern recognition technology. In this subsection, the performances of the combination of feature engineering and classifiers are displayed and analyzed. Four types of acoustic features are extracted, which are time-domain features, FFT-based spectral features, auditory-based spectral features, and spectral envelope features. Four classifiers are adopted, including random forest (RF), k-nearest neighbor (KNN), support vector machine (SVM), and linear discriminant analysis (LDA).

Time-domain features used in this work contain short-term energy (STE), pitch, and fluency features. The STE feature of speech signals reflects the amplitude variation, and the pitch indicates the vocal cords vibration in the pronunciation process. The fluency feature can reflect the degree of coherence in expression. Considering the reduced syntactic complexity and abnormal pauses in schizophrenic speech, five fluency features (total recording time, the total length of voice segments, the ratio of voice segments, max duration of pauses, mean length of syllables) are employed to construct a feature set.

FFT-based features refer to the features computed by the STFT. In this work, two FFT-based features (spectrogram and long-term average spectrum (LTAS)) are adopted in this work. The LTAS describes the resonance characteristics by computing the short-term Fourier magnitude spectra [45], which have shown promising performance in speech sentiment analysis and pathological speech analysis [46–48].

Auditory-based features are proposed to simulate the clinical diagnosis. Schizophrenia is diagnosed by clinicians through a comprehensive evaluation of speech and behaviors. Therefore, speech signals are necessary to be analyzed by combining with human auditory characteristics. In this study, MFCC and its modification, Gammatone cepstral coefficient (GTCC) [49], are extracted to detect schizophrenia. The MFCCs and GTCCs are computed using a series of filters that are designed according to the frequency response characteristics of the human auditory system.

The spectral envelope feature is also commonly used to describe the vocal tract characteristics in speech production. In this work, LP and its deformations, stabilized weighted linear prediction (SWLP) [50] and extended weighted linear prediction (XLP) [51], are tested on the schizophrenic speech data set. The SWLP is an improved WLP that is proposed to model speech by applying the temporal weighting of the square of the residual signal [50]. The XLP is a further generation of WLP and SWLP methods, which allows temporal weighting on a finer time scale [51]. The SWLP and XLP have performed well on the speech recognition tasks and pathological speech detection [52, 53].

The features mentioned above combined with four classifiers are tested on schizophrenic speech data set. The overall performances are listed in Table 4 using accuracy, precision, recall, and F1-score. The bold font in Table 4 represents the highest value in each type of features using different classifiers. It can be seen that fluency feature, spectrogram, GTCC, and XLP achieve the highest F1-score in its corresponding feature group. When compared the results in Tables 3 and 4, it can be seen that the proposed Sch-net has a better performance than the models based on feature engineering and classifiers.

**Table 4:** Performance of feature engineering and classifiers on schizophrenic speech detection

Classifier		Feature									
		Time-domain feature			FFT-based spectral feature		Auditory-based spectral feature		Spectral envelope feature		
		STE	Pitch	Fluency feature	LTAS	Spectrogram	MFCC	GTCC	LP	SWLP	XLP
RF	Accuracy	0.7686	0.5935	**0.8213**	0.6464	**0.8972**	0.8043	**0.8791**	0.9245	0.9377	**0.9423**
	Precision	0.6251	0.5847	**0.8281**	0.6052	**0.8946**	0.7818	**0.8487**	0.9055	**0.9319**	0.9282
	Recall	0.7126	0.5754	**0.8103**	0.5545	**0.9103**	0.8577	**0.9289**	0.9549	0.9466	**0.9644**
	F1-score	0.6306	0.6322	**0.8133**	0.5513	**0.8972**	0.8144	**0.8856**	0.9280	0.9391	**0.9453**
KNN	Accuracy	**0.7723**	0.5385	0.7390	0.7504	**0.8974**	0.8626	**0.8753**	0.9204	0.9287	**0.9375**
	Precision	0.6410	0.5308	**0.7050**	0.6489	**0.8566**	0.8977	**0.9043**	0.8939	0.9117	**0.9196**
	Recall	0.6063	0.5597	**0.7985**	0.6152	**0.9636**	0.8312	**0.8494**	**0.9640**	0.9549	0.9636
	F1-score	0.6123	0.5382	**0.7418**	0.6211	**0.9046**	0.8591	**0.8700**	0.9257	0.9315	**0.9398**
SVM	Accuracy	**0.7905**	0.5172	0.7746	0.7358	**0.9024**	0.8625	**0.8929**	0.9164	0.9291	**0.9334**
	Precision	0.6447	0.5087	**0.7657**	0.6556	**0.8741**	0.8555	**0.8762**	0.8980	**0.9183**	0.9126
	Recall	0.5999	0.4767	**0.7875**	0.5435	**0.9549**	0.8929	**0.9198**	0.9470	0.9466	**0.9636**
	F1-score	0.6155	0.4644	**0.7627**	0.5813	**0.9091**	0.8689	**0.8960**	0.9206	0.9317	**0.9356**
LDA	Accuracy	**0.7858**	0.5087	0.7452	0.7314	**0.8385**	0.8887	**0.9198**	0.9026	0.9069	**0.9109**
	Precision	0.6447	0.4625	**0.7083**	0.6622	**0.8053**	0.9394	**0.9479**	0.8963	**0.9053**	0.8868
	Recall	0.5898	0.5391	**0.7522**	0.5380	**0.9095**	0.8474	**0.8933**	0.9198	0.9111	**0.9462**
	F1-score	0.6093	0.4710	**0.7104**	0.5821	**0.8498**	0.8807	**0.9161**	0.9060	0.9079	**0.9146**

#### Time-domain feature

As shown in Table 4, the F1-score of schizophrenic speech detection using the STE reaches 0.6306. Owing to the difficulty in expression for schizophrenic patients, the intensity of schizophrenic speech tends to be lower than that of controls. The STE feature can describe the intensity of speech, but it may be influenced by the different distances between the recording equipment and speakers. Thus, the performance of the STE feature is not as good as the fluency feature.

Though studies [28, 30–32] have proved that there are significant differences in pitch between schizophrenic speech and normal speech, the pitch gains the worst performance among time-domain features. The results are consistent with the results in [30, 37], in which the distribution of pitch shows no significant differences between the two groups.

Fluency feature performs well on the schizophrenic speech detection, owing to the disordered thought and language impairments of patients [54]. The cognitive impairment also contributes to the incoherence of speech.

#### FFT-based spectral feature

The LTAS achieves 62.11% accuracy on the schizophrenic speech data set. The LTAS is calculated as the average of a spectrogram, reflecting the spectrum of glottal source and vocal tract [55]. Results in [30] have shown that schizophrenic speech has lower variations in energy than normal speech. The unexpected accuracy using LTAS may be caused by the average operation that eliminates the differences in variations between two groups.

The spectrogram achieves better performance than the LTAS, which is the time-frequency representation of speech. It not only contains the energy distribution in frequency bands but also reflects the pitch and formant information. It has been proven that schizophrenic speech have less variability in pitch and voice intensity, smaller range of second formant than normal speech [28, 30–32]. Thus, the spectrogram covers more effective features for discriminating patients from controls than the LTAS does.

#### Auditory-based spectral feature

The GTCC achieves a better performance than the MFCC on the schizophrenic speech detection task, which is caused using different auditory filters. The MFCC is computed based on a series of triangular bandpass filters with equal bandwidth. The GTCC employs the Gammatone filters to model the human auditory response, which are with equivalent rectangular bandwidth [56]. The use of Gammatone filters minimizes the loss of spectrum information and increases the correlation among the outputs of the filters [56]. Therefore, the GTCC contains more effective information to detect schizophrenia than the MFCC.

#### Spectral envelope feature

The F1-scores of schizophrenic speech detection using LP, SWLP and XLP are above 0.9. The SWLP and XLP have slightly better results than LP. The results of spectral envelop features are gained when the order of LP is set as 38 [57, 58]. Results in [32, 37] have shown that formant is an indicator to distinguish schizophrenic speech from controls. The LP reflects the characteristics of the vocal tract, such as the frequency of formants. However, the LP analysis relies on the excitation signal, which is usually affected by the harmonics. The SWLP reduces the effect by composing the temporal weights on the closed-phase interval of the glottal cycle [53]. In addition, the XLP improves the time scale on the spectral envelop by weighting each lagged speech signal separately [53]. The SWLP and XLP highlight the formant information that can be used to distinguish patients from controls. Thus, the SWLP and XLP achieve better performance on classifying schizophrenia and controls than the LP.

### Comparison with classic deep neural networks

**Fig. 2:**
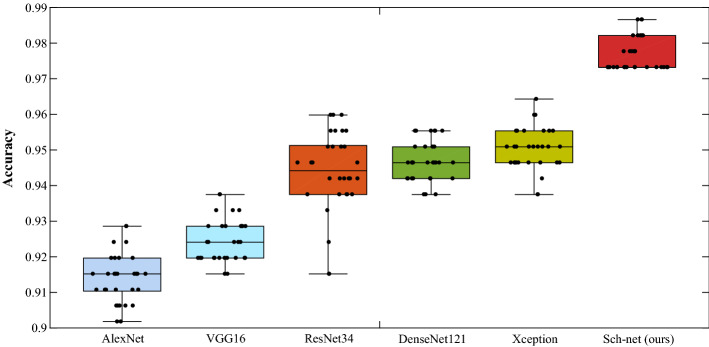
Box plots of accuracy for classifying schizophrenic speech and controls using five neural networks and Sch-net

In this subsection, comparisons between five neural networks and our model are conducted. The five networks are AlexNet [59], VGG16 [60], ResNet34 [61], DenseNet121 [62], and Xception [63], which are commonly used for speech recognition and classification tasks [64–68]. AlexNet [59] is the winner of the ImageNet Large Scale Visual Recognition Challenge in 2012, which reduces overfitting and controls the model complexity of the FC layers using dropout. VGG16 [60] is a good benchmark architecture for classification tasks, which is consisted of 13 Conv layers, 3 FC layers, and 5 pooling layers. ResNet34 [61] is introduced to alleviate the degradation problem caused by increasing stacked layers via adding shortcut connections. To reduce the impact on vanishing gradient, the feed-forward fashion in the connection between each layer to every other layer is used in DenseNet121 [62]. DenseNet121 also can strengthen the propagation of features and reduce the number of parameters [62]. To obtain fast convergence and good performance on the model’s expressive ability, Xception [63] replaces the inception modules with depthwise separable convolutions in deep CNN. Table 5 lists the 95% CIs for seven metrics of classifying schizophrenic speech and normal speech using the five deep neural networks and our method. Fig. 2 presents the box plots of the classification accuracies for the models.

**Table 5:** Performance of schizophrenic speech detection using classic deep neural networks and the proposed Sch-net

Evaluated indicators	95% CI					
	AlexNet	VGG16	ResNet34	DenseNet121	Xception	Sch-net (ours)
Accuracy	0.9272	0.9247	0.9439	0.9469	0.9503	**0.9768**
	(0.9249,0.9295)	(0.9225,0.9269)	(0.9398,0.9480)	(0.9449,0.9489)	(0.9482,0.9524)	**(0.9739,0.9797)**
Precision	0.9279	0.8937	0.9074	0.9555	0.9462	**0.9639**
	(0.9226,0.9333)	(0.8900,0.8973)	(0.9024,0.9124)	(0.9516,0.9594)	(0.9421,0.9503)	**(0.9585,0.9693)**
Recall	0.9268	0.9643	0.9890	0.9375	0.9551	**0.9908**
	(0.9251,0.9285)	(0.9643,0.9643)	(0.9822,0.9958)	(0.9375,0.9375)	(0.9545,0.9556)	**(0.9898,0.9918)**
F1-score	0.9273	0.9276	0.9463	0.9464	0.9506	**0.9771**
	(0.9252,0.9293)	(0.9257,0.9295)	(0.9423,0.9503)	(0.9445,0.9483)	(0.9486,0.9526)	**(0.9743,0.9799)**
Sensitivity	0.6399	0.8795	0.9798	0.9262	0.9482	**0.9914**
	(0.6244,0.6554)	(0.8715,0.8875)	(0.9725,0.9870)	(0.9244,0.9280)	(0.9409,0.9556)	**(0.9863,0.9964)**
Specificity	0.8747	0.9268	0.9399	**0.9938**	0.9646	0.9738
	(0.8564,0.893)	(0.9164,0.9372)	(0.9331,0.9467)	**(0.9910,0.9965)**	(0.9567,0.9724)	(0.9656,0.9820)
AUC	0.7935	0.9447	0.9888	0.9908	0.9924	**0.9978**
	(0.7868,0.8003)	(0.9422,0.9472)	(0.9855,0.9921)	(0.9899,0.9917)	(0.9912,0.9936)	**(0.9965,0.9990)**

As shown in Table 5 and Fig. 2, the accuracies of schizophrenic speech detection using AlexNet and VGG16 are 92.72% (95% CI: 92.49–92.95%) and 92.47% (95% CI: 92.25–92.69%), respectively. The depth of AlexNet and VGG16 is shallow, contributing to the insufficient information in feature maps. ResNet34 achieves 94.39% (95% CI: 93.98–94.80%) accuracy on the schizophrenic speech data set, owing to the introduction of the residual module. DenseNet121 and Xception gain slightly better results than ResNet34, owing to the networks not only adopt the shortcut connections but also utilize dense connection/depthwise separable convolutions to make more efficient use of model parameters. The proposed Sch-net in this work achieves a better performance than the five networks, because it can gain the local and global features simultaneously via CBAM and skip connections. The feature map contains more abundant information to better distinguish schizophrenia from controls.

### Network visualization using Grad-CAM

**Fig. 3:**
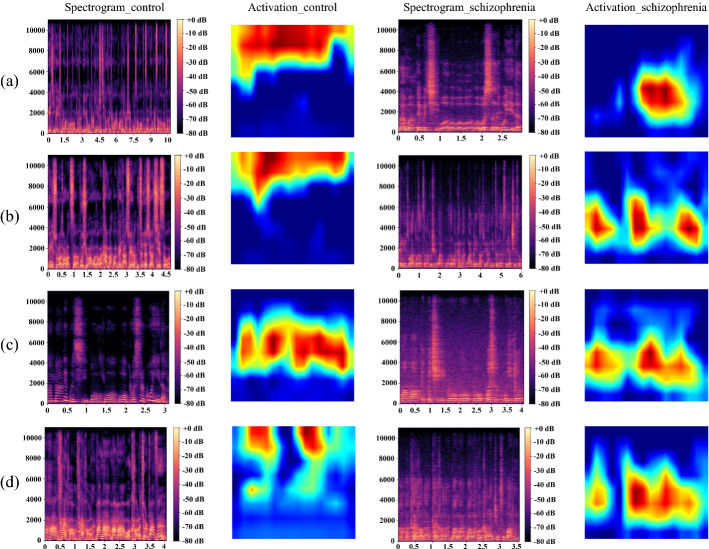
Spectrogram and corresponding activation map of normal speech and schizophrenic speech in four emotions. **a** The spectrogram and corresponding activation map of normal speech and schizophrenic speech in calm emotion. **b** The spectrogram and corresponding activation map of normal speech and schizophrenic speech in anger emotion. **c** The spectrogram and corresponding activation map of normal speech and schizophrenic speech in fear emotion. **d** The spectrogram and corresponding activation map of normal speech and schizophrenic speech in happiness emotion

In recent years, deep learning methods have already achieved high accuracy that approaches the manual diagnosis accuracy in many fields through improving the computing capabilities and expanding the data set. It can simplify and speed up the diagnosis, and reduce the workload of doctors. However, the process of generating predicted labels from input data is still uninterpretable. To make the decision-making process in deep learning transparent, this work applies the Grad-CAM [69] to Sch-net using speech samples from schizophrenic group and healthy group. Grad-CAM is a visualization method to show the importance of each neuron for the classification using the gradient information in the last Conv layer [69]. The Grad-CAM highlights the more discriminative parts as brighter regions in the heatmap. We attempt to consider how the Sch-net works on making good use of features, through observing the spectrogram and activation maps. In this subsection, the input spectrogram and its corresponding activation map generated in the last Conv layer of normal speech and schizophrenic speech are shown in Fig. 3.

In Fig. 3, spectrograms of normal speech and schizophrenic speech are shown in a and c, respectively. Activation maps of normal speech and schizophrenic speech are depicted in b and d. The brighter region in the spectrogram means more energy concentrated, and that in the activation map means larger weight located.

As shown in Fig. 3a, c, schizophrenic speech and normal speech have different distributions of concentrated energy in the spectrogram. Through the horizontal comparison, two findings of two groups can be seen in this figure, which can be listed as follows:

1. The energy concentration in the frequency domain of schizophrenic speech is almost below 5000 Hz, while normal speech has a wider range of energy concentration bands, that can be extended from 8000 to 10,000 Hz. Blunted affect is a typical symptom in schizophrenia [70]. Patients with negative symptoms may speak with a dull monotone voice [71], resulting in a small range of the energy concentration region. While healthy controls have a more flexible emotional expression. The angry, fearful and happy speech exhibit a higher intonation, faster speed rate, and more energy in higher frequencies [72]. And the sad speech changes slowly and has high energy concentration in lower frequencies [73]. Thus, normal speech has a wider range of energy distribution than schizophrenic speech.
2. It can be seen that schizophrenic speech and normal speech both have concentrated energy region and apparent formant horizontal stripes in the low-frequency bands below 2000 Hz. The difference between the two groups is the shape of formant horizontal stripes. For schizophrenic speech, the stripes are almost continuous, which is inconsistent with the energy distribution characteristics of vowels and consonants. The vowels have energy concentration in both low- and high-frequency range [74]. The unvoiced consonants mainly have high-frequency energy components, and they rarely have formants [75]. According to the texture used in this work, the continuous-time speech signals comprise both vowels and consonants. Therefore, there are supposed to show a short disappearance of formant horizontal stripes on the spectrogram. It can be guessed that the continuous stripes in the spectrogram of schizophrenic speech may be caused by the incorrect placement of articulators during speech production. The wrong articulation process leads to the unvoiced consonants are produced as voiced consonants.

Observing both the spectrogram and its corresponding activation map in Fig. 3, it can be seen that the Sch-net can capture the features in high-frequency bands for normal speech, and can give larger weights to the features in low-frequency bands for schizophrenic speech. The results of Sch-net are consistent with human visual perception, which is difficult to achieve using the models based on feature engineering. The Sch-net has excellent learning ability to extract features, and it achieves better performances on schizophrenic speech detection than traditional feature engineering models adopted in this work.

### Further validation of the proposed Sch-net using LANNA children speech database

Schizophrenia is a neurodevelopmental disorder affecting the language expression of patients [76]. SLI, also termed development dysphasia, is described as a neurological disorder of the brain [77–80]. Patients with SLI exhibit delayed language acquisition [81], slower linguistic processing [82], and difficulties in grammar or specific subcomponents of grammar [83, 84]. To further validate model effectiveness and generalization, the Sch-net is tested on LANNA children speech database [85] for the classification of patients with SLI and healthy controls in this subsection.

LANNA children speech database [85] is the first and only publicly open speech corpora for children with SLI, which comprises 2173 speech signals from 54 children with SLI (aged from 6 to 11 years) and 1680 speech signals from 44 controls (aged from 6 to 10 years). This data set is composed of 13 parts: vowels, consonants, syllables, six types of words, sentences, auditory differentiation, and description of the picture. Audios were recorded in a schoolroom and a consulting room using Dictaphone, MD and microphone. The background noise in natural environments affects the quality of speech signals, leading to difficulties in speech signal processing.

Previous studies [85–91] had demonstrated that speech can be viewed as a symbol of diagnosing SLI. In [85–87], 1582 acoustic features were extracted from 34 low-level descriptors and its 21 statistical functionals. The features were given as inputs of the SVM, achieving 96.94% accuracy on the LANNA children speech database. In [88], Gaussian posteriorgrams trained on MFCC features were employed to discriminate patients with SLI and healthy controls. The kernel extreme learning machine were trained with the speech signals, and it performed an accuracy of 99.41% on the test data. Apart from MFCC, in [89], Tonnetz and Chroma were calculated, combined with SVM, RF and Recurrent Neural Network to detect SLI. The Tonnetz and Chroma reached accuracies of 70% and 71%, respectively. In the four studies [85–89], high accuracies had been achieved for speaker-dependent classification.

In contrast, some methods were proposed for speaker-independent classification in [90, 91]. The top-20 LPC features were selected from 408 LPCs using Mann–Whitney* U* test and Spearman’s correlation in [90], which achieved an accuracy of 97.90% on the SLI detection task. In [91], a feed-forward neural network was proposed for classifying patients with SLI and healthy controls. The glottal features and MFCCs were adopted as the inputs of the network and the classification accuracy reached up to 98.82%.

In this subsection, fivefold cross-validation is employed. SLI data set is divided with 80% for training and 20% for testing. Table 6 gives the classification results using state-of-the-art methods, deep neural networks and the proposed Sch-net. As can be seen, our method outperforms the classic deep neural network and state-of-the-art methods. The proposed Sch-net can extract discriminant features of speech signals for classifying healthy individuals and those suffered from SLI.

**Table 6:** Results of SLI detection using state-of-the-art methods, classic deep neural networks and the proposed Sch-net

Method		Accuracy	Precision	Recall	F1-score
State-of-the-art method	Grill [85–87]	0.9694	1.0000	0.9474	0.9730
	Ramarao [88]	0.9941	**-**	**-**	**-**
	Slogrove [89]	0.9800	0.9900	0.9900	0.9900
	Sharma [90]	0.9790	-	-	**-**
	Reddy [91]	0.9882	**-**	**-**	**-**
Deep Neural Network	AlexNet	0.9132	0.9585	0.8810	0.9181
	VGG16	0.9230	0.9897	0.8787	0.9309
	ResNet34	0.9329	0.9489	0.9286	0.9386
	DenseNet121	0.9461	0.9397	0.9643	0.9518
	Xception	0.9622	0.9514	0.9863	0.9685
	Sch-net (our)	0.9952	0.9979	0.9937	0.9958

## Conclusions

In this work, we propose an Sch-net neural network for automatic detection of schizophrenia based on speech signals. This is the first work to detect schizophrenic speech using deep learning techniques. The Sch-net is performed using a set of convolutional layers. The global and local features are merged using skip connections, and the effective features are highlighted by using CBAM. In the experiments, the advantages of embedding the SC and CBAM into the backbone architecture are verified in ablation studies. The proposed model can learn the differences in speech patterns between patients and healthy controls automatically, avoiding the requirements of domain knowledge for designers. The comparisons with the models based on feature engineering and classic deep neural networks are conducted on a schizophrenic speech data set that contains 28 schizophrenic patients and 28 healthy controls. The experimental results show that the Sch-net has achieved 97.68% accuracy. In addition, we visualize how the model performs on extracting features given an input spectrogram. The Grad-CAM heatmaps show the region that the Sch-net focuses on is consistent with human visual perception. Finally, the proposed method is further validated on the open access LANNA children speech database, achieving 99.52% accuracy on classifying patients with SLI and healthy controls.

The clinical diagnosis of schizophrenia is made by expertise psychiatrists based on a full psychiatric assessment, which depends on the experience of psychiatrists. The reports are often affected by the patients’ retrospective recall bias and cognitive limitations. Moreover, the diagnosis is high-cost and time-consuming, and the high patients-to-clinicians ratio leads to the heavy workload of clinicians. The proposed model can serve as an aid to psychiatrists for the diagnosis of schizophrenia. It can automatically discriminate schizophrenic speech from controls, which may be helpful to the preliminary screening for schizophrenia. In addition, it can provide low-cost and long-term monitoring for patients with schizophrenia, and reduce the workload of clinicians.

Our work still has several limitations. First, the proposed model can only achieve the classification of patients and healthy controls, but cannot assess disease severity. Second, the generalization of the proposed model needs to be further verified. Future work will seek to perform extensive validation using a larger number of databases that record speech signals of patients with psychological/neurological disorders.

## Methods

**Fig. 4:**
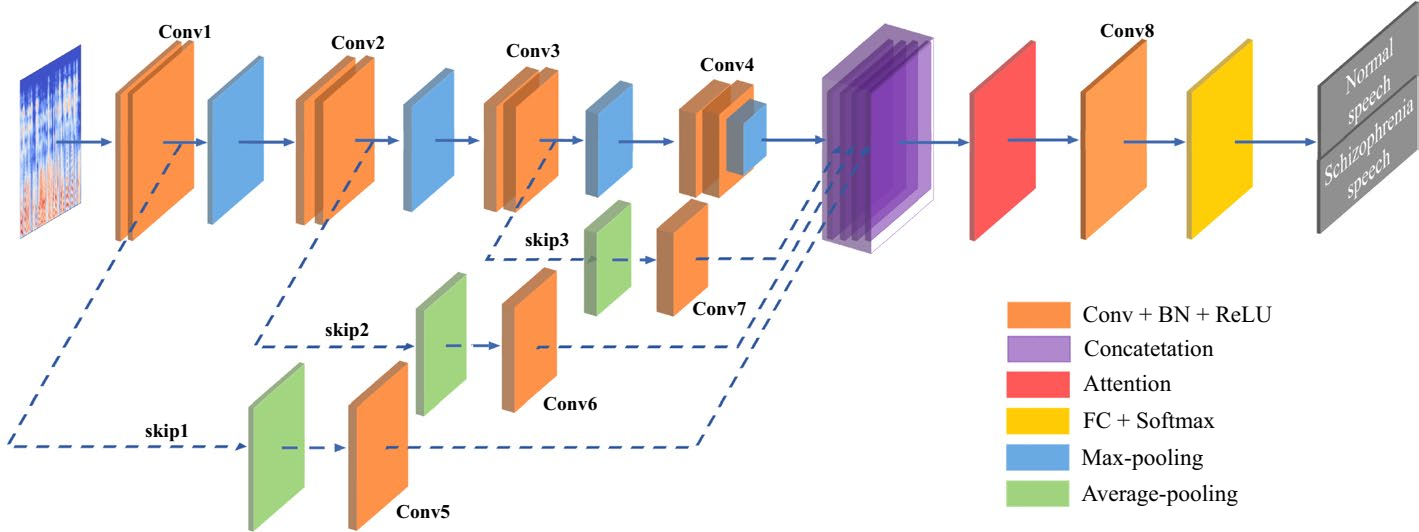
Architecture of Sch-net for automatic schizophrenic speech detection

In this work, we have developed a CNN-based architecture, termed Sch-net, to classify schizophrenic speech and normal speech. The architecture of the proposed model is depicted in Fig. 4. The input is the spectrogram containing time–frequency domain information of speech signals. There are 12 convolutional (Conv) layers, 6 pooling layers, skip connections, an attention module and a fully connected (FC) layer. The FC layer is composed of two hidden layers. A softmax function is employed to the output of the FC layer, and the output of the softmax is the classification result of speech samples. The backbone network and two essential components (skip connections and CBAM) of Sch-net are described below.

### Backbone network of Sch-net

**Fig. 5:**
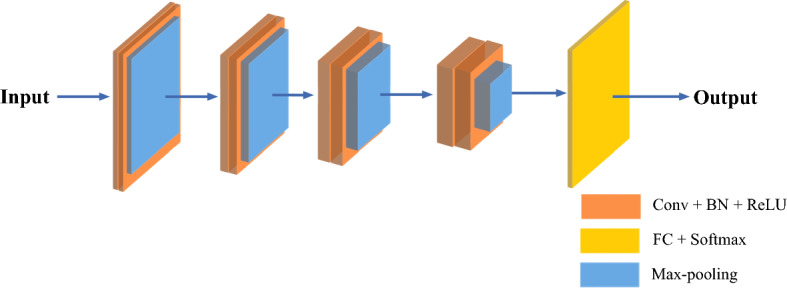
Diagram of the backbone network of Sch-net

The backbone network of Sch-net shown in Fig. 5 is consisted of Conv layer, pooling layer, batch normalization (BN) component, rectified linear unit (ReLU) and FC layer. When spectrogram is given as the input of Sch-net, local features in spectrogram are extracted via Conv layer. The dimension of features and the amount of computation are reduced in the pooling layer via max pooling operation [92]. As the number of hidden layers increases, the network would suffer from the gradient vanishing and exploding problems. To address these problems, the BN layer and ReLU activation function are adopted. The introduction of BN components can also speed up the convergence, cut down the regularization process, and enable to train the network with a larger learning rate [93, 94]. ReLU is a typical activation function in deep learning, which works better than sigmoid and tanh activation functions in speech recognition tasks [95, 96]. It removes the negative values in the feature map and is identity for all positive values [97]. The networks can be trained effectively using the ReLU even without pre-training [98]. At the end of the network, the FC layer and softmax function are employed to achieve the classification task. The FC layer is essential to transfer CNN-based network visual representation in classification tasks [99]. Each node in the FC layer is connected to all activation values in the previous layers.

### Skip connections

**Fig. 6:**
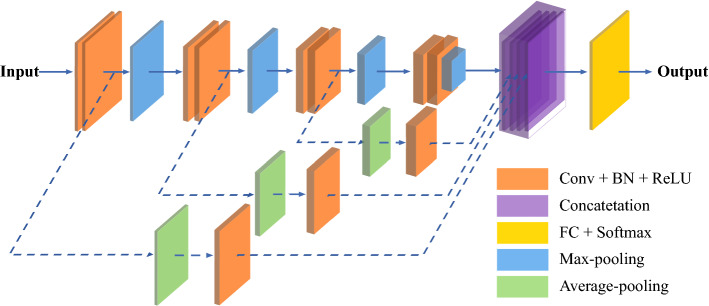
Diagram of the backbone network + skip connections of Sch-net

The backbone network of Sch-net can extract the local features in spectrogram via shallow layers and max-pooling operation. There is no evidence that schizophrenic patients have a special pattern in pronunciation or schizophrenic speech has prominent local acoustic features. Thus, global features are supposed to be extracted for schizophrenic speech detection. To retain more original and global information in the input feature map, average pooling operation and skip connections are added to the backbone network of Sch-net. Average pooling considers all the values in the batch that has an equal size with the pooling kernel. Skip connection allows the low-level feature map to skip some layers in the neural network and merge with high-level feature maps [100]. This connection combines the features after max-pooling and average-pooling, superimposed into a feature. Skip connections expand the dimensions of features in the network, providing more information for the classification task. The diagram of the backbone network of Sch-net with skip connections is given in Fig.6.

### Attention mechanism

The output of skip connections contains low-level and high-level features. To emphasize the meaningful features and suppress the unnecessary ones for the classification task, an attention module is added to the backbone network. The output of the attention module is calculated as the weighted sum of the input values [101]. The bigger weights mean the more attention would be paid to the input. This work adopts a lightweight and general module, CBAM [102], to improve the performance of the network. The CBAM is composed of channel and spatial attention modules [102]. The channel attention module focuses on “what” is the effective part in the feature map by utilizing max-pooling and average-pooling with a shared network [102]. The spatial attention module tells “where” to focus or suppress by employing a Conv layer [102]. The CBAM used in the Sch-net can effectively refine the intermediate feature map with negligible computation and overheads.

## Data Availability

Not applicable.

## Abbreviations

CNN: Convolutional Neural Network
CBAM: Convolutional Block Attention Module
MFCC: Mel-frequency Cepstral Coefficient
LP: Linear Prediction
LPC: Linear Prediction Coefficient
Conv: Convolutional
FC: Fully connected
BN: Batch Normalization
ReLU: Rectified Linear Unit
FFT: Fast Fourier Transform
RF: Random Forest
KNN: K-Nearest Neighbor
SVM: Support Vector Machine
LDA: Linear Discriminant Analysis
STE: Short-term Energy
LTAS: Long-term Average Spectrum
GTCC: Gammatone Cepstral Coefficient
WLP: Weighted Linear Prediction
SWLP: Stabilized Weighted Linear Prediction
XLP: Extended Weighted Linear Prediction
